# Effect of a Neonatal Resuscitation Course on Healthcare Providers’ Performances Assessed by Video Recording in a Low-Resource Setting

**DOI:** 10.1371/journal.pone.0144443

**Published:** 2015-12-11

**Authors:** Daniele Trevisanuto, Federica Bertuola, Paolo Lanzoni, Francesco Cavallin, Eduardo Matediana, Olivier Wingi Manzungu, Ermelinda Gomez, Liviana Da Dalt, Giovanni Putoto

**Affiliations:** 1 Department of Women and Children Health, School of Medicine, Padua University, Azienda Ospedaliera di Padova, Padua, Italy; 2 Doctors with Africa CUAMM, Padua, Italy; 3 Independent Statistician, Padua, Italy; 4 Department of Obstetrics and Gynecology, Beira Central Hospital, Beira, Mozambique; 5 Pediatric Department, Beira Central Hospital, Beira, Mozambique; Klinikum rechts der Isar - Technical University Munich - TUM, GERMANY

## Abstract

**Background:**

We assessed the effect of an adapted neonatal resuscitation program (NRP) course on healthcare providers’ performances in a low-resource setting through the use of video recording.

**Methods:**

A video recorder, mounted to the radiant warmers in the delivery rooms at Beira Central Hospital, Mozambique, was used to record all resuscitations. One-hundred resuscitations (50 before and 50 after participation in an adapted NRP course) were collected and assessed based on a previously published score.

**Results:**

All 100 neonates received initial steps; from these, 77 and 32 needed bag-mask ventilation (BMV) and chest compressions (CC), respectively. There was a significant improvement in resuscitation scores in all levels of resuscitation from before to after the course: for “initial steps”, the score increased from 33% (IQR 28–39) to 44% (IQR 39–56), p<0.0001; for BMV, from 20% (20–40) to 40% (40–60), p = 0.001; and for CC, from 0% (0–10) to 20% (0–50), p = 0.01. Times of resuscitative interventions after the course were improved in comparison to those obtained before the course, but remained non-compliant with the recommended algorithm.

**Conclusions:**

Although resuscitations remained below the recommended standards in terms of quality and time of execution, clinical practice of healthcare providers improved after participation in an adapted NRP course. Video recording was well-accepted by the staff, useful for objective assessment of performance during resuscitation, and can be used as an educational tool in a low-resource setting.

## Introduction

Each year about 6.6 million children worldwide under the age of 5 die and of these 44% are newborns. According to the World Health Organization, about a quarter of these newborns die in the first 24 hours. To these, we must add the 3 million stillbirths recorded annually [1–3].

In 2012, the neonatal mortality rate in Mozambique was estimated at 34/1000 live births. The rate of stillbirth was 28/1000 total births. Neonatal deaths constituted 35% of an estimated 85000 deaths under five years of age [4].

Intrapartum-related events, previously called “birth asphyxia”, account for a quarter of neonatal deaths [5]. Initiation of breathing is critical in the physiologic transition from intrauterine to extrauterine life. Approximately 85% of babies born at term will initiate spontaneous respirations within 10 to 30 seconds of birth, an additional 10% will respond during drying and stimulation, approximately 3% will initiate respirations after positive-pressure ventilation (PPV), 2% will be intubated to support respiratory function, and 0.1% will require chest compressions (CC) and/or epinephrine to achieve this transition [6].

Therefore, training programs on neonatal resuscitation for all health workers involved in the management of the newborn at birth are a critical priority to improve neonatal survival [7–11]. Previous studies aimed at assessing the effect of such training programs showed an improvement in the knowledge and skills of the healthcare providers and their degree of comfort in the execution of interventions in high as well as low-resource settings. [12,13]. This improvement, however, did not always translate into a sustainable and long-term improvement in clinical practice in the delivery room [13–15]. This problem could be due to several factors such as poor quality of clinical practices, lack of leadership and supervision, limited availability of supplies and equipment, and low involvement of health workers in organizational and training programs.

Other studies showed that implementing educational programs on neonatal resuscitation in low-resource settings resulted in a reduction of the rate of stillbirths without substantial reductions in the neonatal mortality rate [16]. Therefore, there is a need to confirm that the knowledge and skills taught are consistently and reliably applied during actual clinical practice.

Recently, video recording has been used as a means of evaluating neonatal resuscitation performance of health caregivers in high-resource settings [17–19]. These studies documented a significant number of deviations from the guidelines [18,19]. To date, there have been no reports on the use of videotaping as a means of assessing the quality of neonatal resuscitations in low-resource countries. This study was the first to objectively examine the effect of resuscitation training on provider practices in a true clinical low-resource setting. Our aim was to use video recording to compare the performances of health providers in a low-resource setting before and after participation in an adapted neonatal resuscitation program (NRP) course.

## Methods

### Setting

This prospective observational study was conducted at Beira Central Hospital, in the province of Sofala, Mozambique where about 4500 deliveries occur every year. Beira Central Hospital is the referral hospital for a geographical area that covers about 7 million people. This center was selected for the study because it is a level III hospital with large referral services for maternal and neonatal care [4]. The study protocol was approved by the National Committee of Bioethics (Ref. 315/CNBS/13; November, 1, 2013) and by the Minister of Health of the Republic of Mozambique (Ref. 08/GMS/002/2014; January, 7, 2014). Parental consent to record neonatal delivery room management and to use the data was obtained before delivery. Written informed consent was given by parents or caregivers for clinical records to be used in this study. All information, including informed consent and all the material used in the study was written in Portuguese in a clearly understandable form.

### Patients

All neonates who needed resuscitation of any form at birth were included in the study. We defined resuscitation as any intervention by healthcare providers: initial steps in order to initiate spontaneous breathing, bag mask ventilation, and/or chest compressions. Lack of parental consent was the only exclusion criterion.

### Study design

All 16 midwives responsible for immediate postnatal management of the newborns at Beira Hospital participated in the study. Participation was mandatory. The study had 3 phases: a) baseline period (data on 50 resuscitations were collected by video recording); b) intervention period (all birth attendants attended a one-day, adapted Neonatal Resuscitation Program (NRP) course); and c) post-intervention period (data on a further 50 resuscitations were collected). The algorithm of 2010 American Heart Association Guidelines was used for the course [7,10]. The NRP algorithm suggests to start PPV at 30 seconds after birth; in case of heart rate less than 60 beats/min despite 30 seconds of effective PPV, CC have to be administered for about 60 seconds; if, despite CC and effective PPV the heart rate remains less than 60 beats/min, medication administration is recommended [7,10].

The adapted NRP course was held in Portuguese by two neonatologists certified as NRP instructors. The training drew heavily on the NRP training in form [20], but was significantly adapted to the Mozambican setting where resources are limited. These adaptions to the NRP training program have not been previously validated or studied. The course teaches an A (Airway), B (Breathing) and C (Circulation) approach to resuscitation laying down a clear step-by-step strategy for the first minutes of resuscitation at birth. We did not include intubation and drug administration because they were not feasible in this setting. The course was comprised of focused lectures aimed at understanding the approach to resuscitation, skill stations and practical scenario sessions using neonatal manikins (Neonatal Resuscitation Baby, Laerdal, Stavanger, Norway) to develop skills in airway opening, bag-mask ventilation (BMV) and CC.

At Beira Central Hospital, neonatal resuscitation is performed routinely on overhead radiant warmers in the delivery room or in the obstetric operating room. Midwives are responsible for immediate postnatal care of all neonates, including resuscitation. The available equipment consists of gloves, clean towels, wall suction device, suction catheters, bulb suction, a self-inflating bag in combination with two face masks (size 0 and 1) and an oxygen source. Neonatal resuscitation is based on the NRP algorithm with the exclusion of intubation and medication administration [7,20].

### Video recording

Interventions were recorded using a webcam for video monitoring (ENXDVR-4C, Encore Electronics. www.encore-usa.com), consisting of 1 fixed camera installed above the radiant warmers both in the delivery room and in the operating room. The cameras provided a 24 hour video recording without audio. The image was zoomed to show only the newborn and the hands of the resuscitation team. Parents, obstetric procedures and faces of the caregivers were not visible [17]. The video camera displayed a continuous time readout at the bottom of the recorded image allowing timing of performed procedures to the nearest second. All videos were stored on a hard disk and sent to the coordinator center (University of Padua). In order to protect the identities of the subjects and the data, all data about resuscitation date and location were removed, and shipment was insured. A skilled neonatologist–aware of the adapted NRP course but blinded to the pre/post-intervention—evaluated and scored each resuscitation according to a previous study [19]. Evaluation of intubation and medications was not included in the score since these steps are not performed in low-resource settings. The modified scoring system is shown in S1 Table.

A composite score was devised to assign a numerical score for each resuscitation. Two points were awarded for every correct decision and every properly performed procedure. One point was awarded if the intervention was delayed or the technique for a given procedure was inadequate. No points were awarded for indicated procedures that were omitted or for performed procedures that were not indicated. The sum of the awarded points was divided by the total possible points for that level of resuscitation (initial steps, BMV and CC) to obtain a percentage score. The “start time” and “stop time” of each procedure was recorded; the “duration of each procedure” could then be calculated from those times.

Maternal and neonatal data were also recorded in a data collection sheet.

### Statistics

Since there were no prior studies of video recording in evaluating neonatal resuscitation performance and in teaching activity in low-resource settings, the sample size was arbitrarily estimated at 50 resuscitations before the course and 50 after the course. Data were expressed as number and percentage or as median and interquartile range (IQR). Categorical data were compared using the Fisher test, whereas continuous data were compared using the Mann-Whitney test. A p-value of less than 0.05 was considered statistically significant. Statistical analysis was performed using R 2.12 software (R Foundation for Statistical Computing, Vienna, Austria).

## Results

### Adapted NRP course

The adapted NRP course was held on January 31, 2014. The median age of the participants was 30 years (IQR 28–36); they had a median of 7 years (4–10) of experience in the delivery room and they had already participated in 2 adapted NRP courses (IRQ 1–2) in the past.

### Patients

During the baseline period (from Jan 11 to Jan 31, 2014), 50 out of 302 (16.5%) neonates were resuscitated; during the post-intervention period (from Feb 2 to March 6, 2014), resuscitation manoeuvres were performed on 50 out of 466 (10.7%) neonates. Maternal and neonatal characteristics of the 2 groups are reported in Table 1.

**Table 1:** Maternal and neonatal characteristics.

	Before training	After training	p-value
Number of resuscitations	50	50	-
**Maternal data**			
Age, years *	23 (19–29)	23 (19–28)	0,57
Antenatal visits *	4 (3–6)	4 (3–6)	0,93
Previous pregnancies *	3 (1–4)	2 (1–3)	0,53
First pregnancy	17 (34)	21 (42)	0,54
HIV infection	10 (20)	12 (24)	0,81
Mode of delivery			0,42
*cesarean section*	23 (46)	28 (56)	
*vaginal delivery*	27 (54)	22 (44)	
Amniotic fluid:			0,27
*clear*	39 (78)	33 (66)	
*meconium stained*	11 (22)	17 (34)	
Complications	32 (64)	36 (72)	0,52
*Placental abruption*	0	4	
*eclampsia/preeclampsia*	12	16	
*dystocia*	6	10	
*uterine rupture*	2	0	
*other*	8	0	
**Neonatal data**			
Gender male:female	32:18	35:15	0,67
Birth weight, g *	2800 (2200–3000)	2950 (2500–3300)	0,07
Gestational age, weeks *	38 (35–40)	38 (37–40)	0,33
Apgar score at 1' *	5 (1–7)	4 (3–6)	0,49
Apgar score at 5' *	6 (2–8)	6 (4–7)	0,73
Deaths, n	13 (26)	14 (28)	0,99

Data are expressed as n (%) or * median (IQR).

All 100 neonates received the initial steps of resuscitation; of them, 77 and 32 needed BMV and CC, respectively.

### Primary outcome

**Fig 1:**
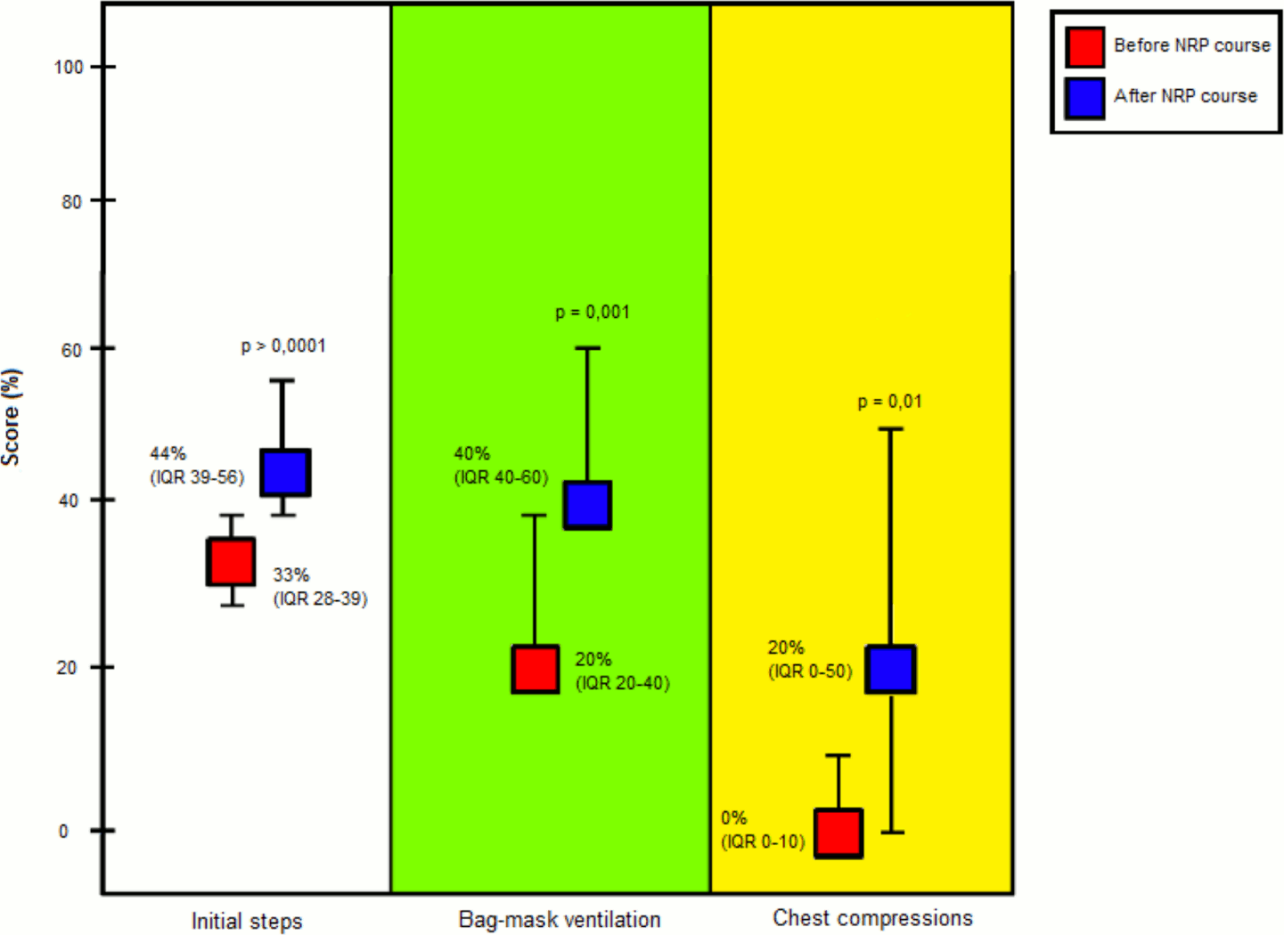
Total scores before and after the course in the three levels (initial steps, BMV and CC) of resuscitation. Data are expressed as median (interquartile range).

Fig 1 shows the percentage scores obtained before and after the course in the three levels of resuscitation.

**Fig 2:**
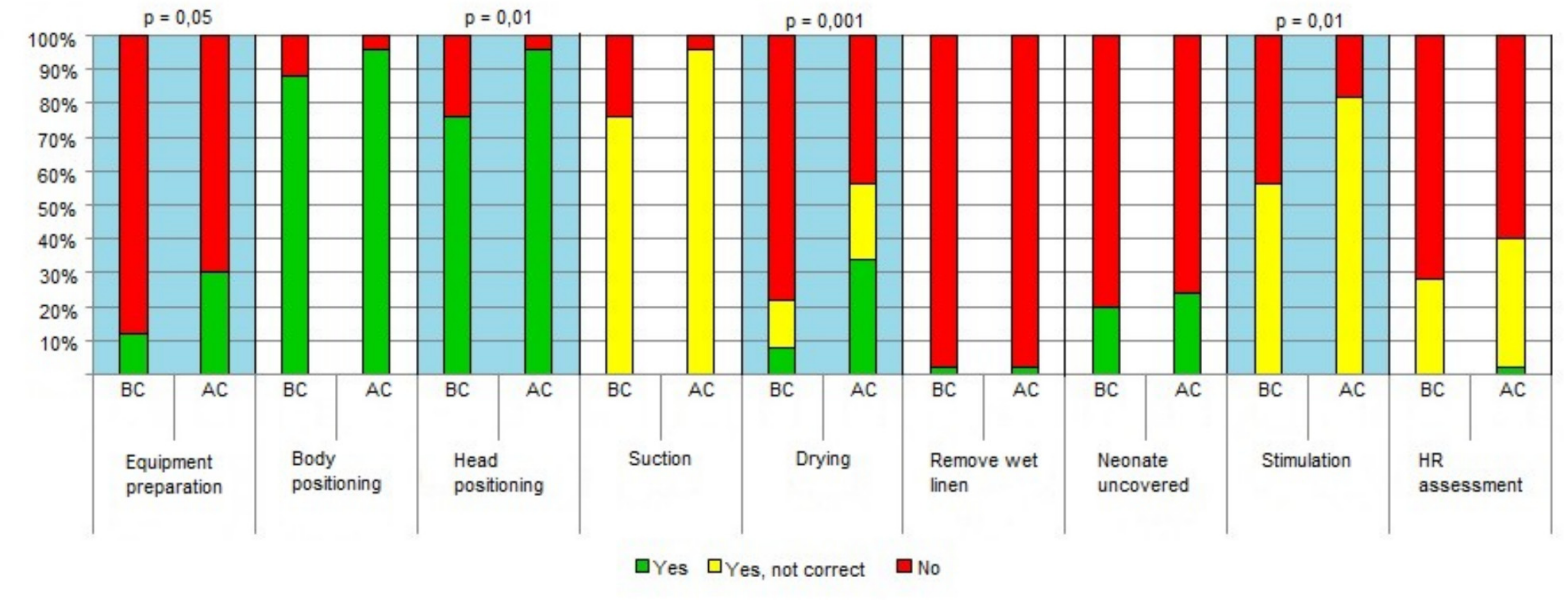
Detailed scores before and after the course in initial steps. (see S1 Dataset). Legend: AC, after the course; BF-before the course; HR- heart rate.

**Fig 3:**
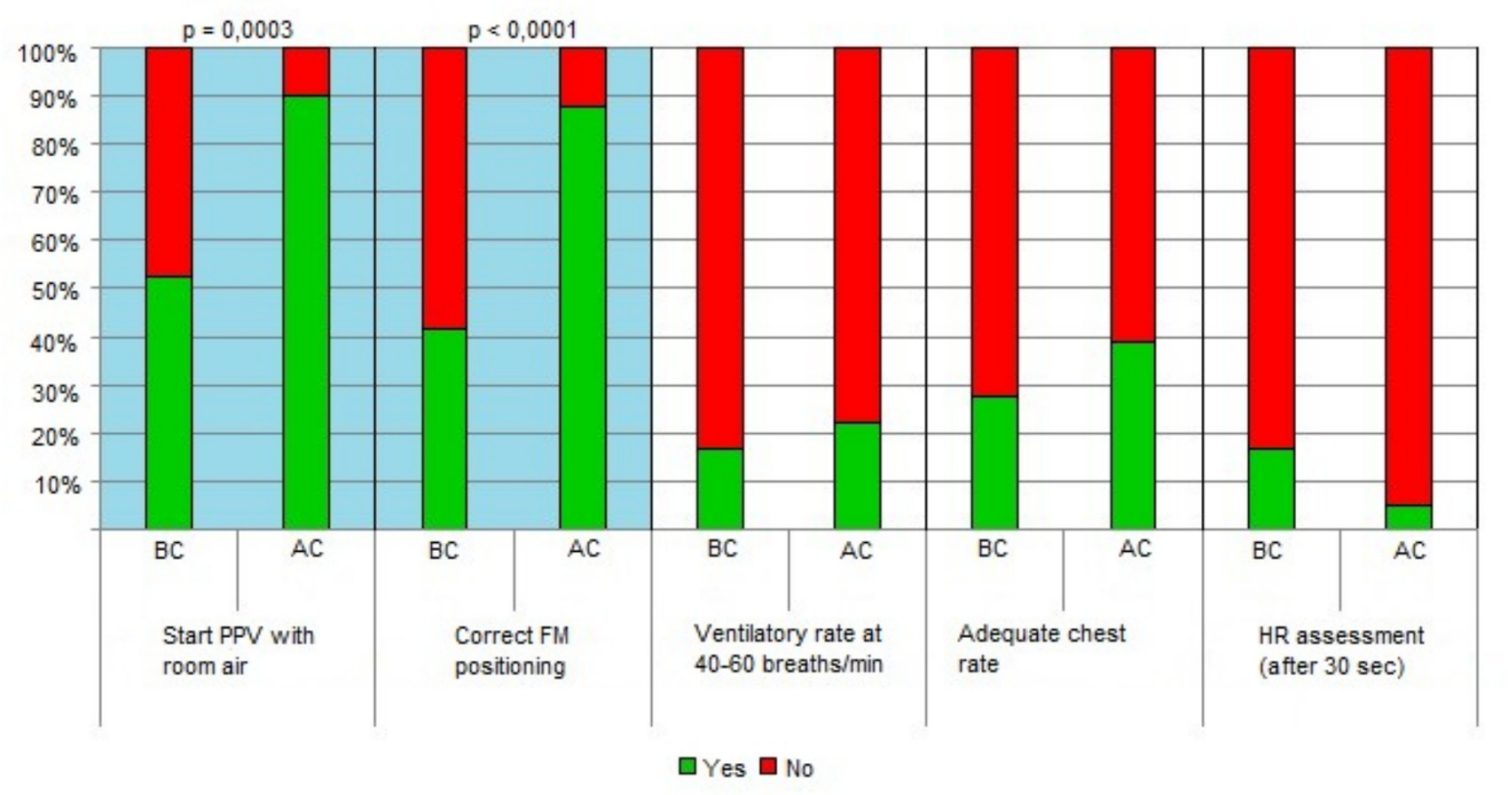
Detailed scores before and after the course in BMV. (see S1 Dataset). Legend: AC, after the course; BF-before the course; FM-face mask; HR- heart rate; PPV- positive pressure ventilation.

**Fig 4:**
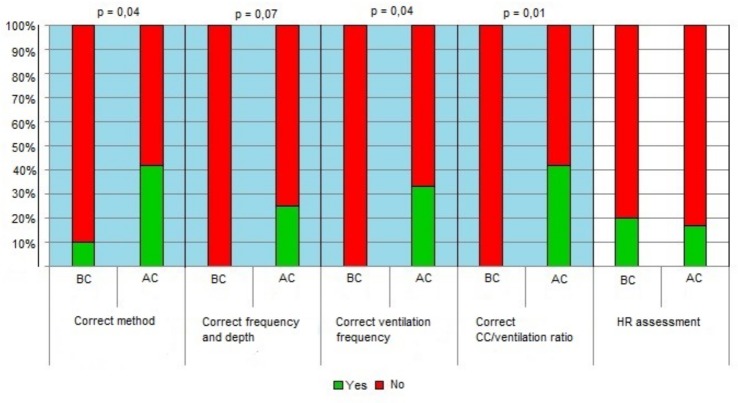
Detailed scores before and after the course in CC. (see S1 Dataset). Legend: AC, after the course; BF-before the course; CC- chest compressions; HR- heart rate.

Within the “Initial steps”, a statistically significant improvement was noted for “preparation of material” (p = 0.05), “positioning of the head” (p = 0.01), “drying” “p = 0.001), and “stimulation” (p = 0.01). (Fig 2) BMV improved in the items “starting ventilation with room air” (p = 0.0003) and “correct positioning of the face mask” (p<0.0001). (Fig 3) All the items included in CC intervention improved with the exception of the frequency of heart rate assessment, which decreased after the course. (Fig 4)

### Timing of interventions

With the exception of “tactile stimulation”, the median start times of all interventions began sooner after the adapted NRP course in comparison to those performed during the baseline period.

**Fig 5:**
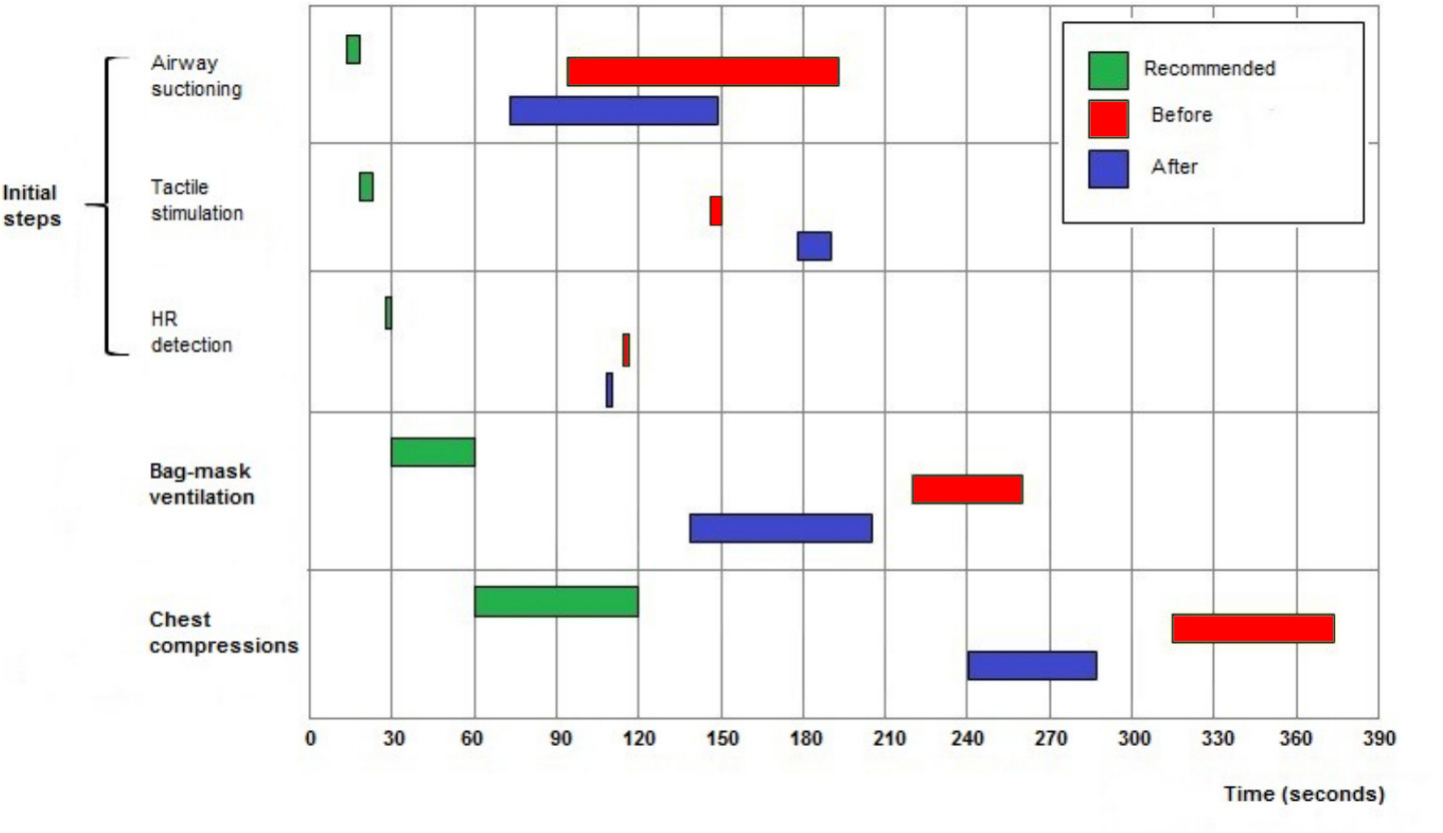
Initiation times and duration of procedures. Data are expressed as medians.

The start time and the duration of each intervention did not adhere to times recommended by the NRP algorithm both before and after the course. (Table 2, Fig 5)

**Table 2:** Time of initiation and duration of procedures.

	Before training	After training	p-value
Total time of procedure	391 (288–680)	366 (263–606)	0,75
Time elapsed from birth to starting resuscitation	21 (0–72)	10 (0–29)	**0,008**
**Suction**			
*Number of interventions*	48	45	-
*Start time of first suction*	94 (64–144)	73 (40–119)	0,07
*Duration of first intervention*	99 (50–158)	76 (46–127)	0,25
*Number of interventions*	1 (1–2)	1 (1–2)	0,16
*Total duration*	125 (60–177)	117 (66–160)	0,53
**Stimulation**			
*Number of interventions*	28	41	-
*Start time of first stimulation*	146 (36–252)	178 (98–239)	0,55
*Duration of first intervention*	4 (3–9)	12 (5–20)	**0,0003**
*Number of interventions*	1 (1–1)	1 (1–2)	0,2
*Total duration*	5 (3–10)	14 (6–28)	**0,0005**
**Heart rate evaluation**			
*Number of interventions*	14	20	-
*Start time of first heart rate evaluation*	116 (30–233)	110 (22–177)	0,86
*Number of interventions*	2 (1–4)	1 (1–3)	0,59
**Bag-mask ventilation**			
*Number of interventions*	36	41	-
*Start time of first bag-mask ventilation*	220 (135–300)	138 (110–226)	**0,03**
*Duration of first intervention*	40 (16–82)	67 (30–134)	0,06
*Number of interventions*	2 81–3)	2 (1–3)	0,64
*Total duration*	83 (32–234)	157 (70–263)	0,24
**Chest compressions**			
*Number of interventions*	20	12	-
*Start time of first chest compressions*	315 (187–466)	241 (137–513)	0,74
*Duration of first intervention*	59 (22–108)	47 (44–97)	0,82
*Number of interventions*	1 (1–2)	2 (1–2)	0,24
*Total duration*	73 (33–121)	105 (52–182)	0,15

Data (seconds) are expressed as median (IQR). *“Number of interventions”* means number of (separate) episodes of that intervention.

## Discussion

In this study, video recording was used to assess the performance of healthcare providers before and after participation in an adapted NRP course in a low-resource setting. Our results show that although resuscitations remained below the recommended standards in terms of quality and time of execution, clinical performance of healthcare providers improved after participation in an adapted NRP course. In addition, our study provides further information on the utility of video recording for human performance assessment, as instituted in a simplified fashion in a low-resource setting.

One-fourth of neonatal deaths each year are attributed are attributed to intrapartum-related events, previously called “birth asphyxia”; of these, nearly all (99%) occur in low and middle income countries. Among the survivors of intrapartum-related events, one million may develop cerebral palsy, learning difficulties, or other forms of disability each year [1–3]. A recent observational study conducted in a Tanzanian rural hospital indicates that asphyxia accounts for a much higher percentage of neonatal deaths in the first week of life (61%) [21], suggesting the urgent need for training programs on neonatal resuscitation for all healthcare workers involved in the management of newborns at birth. Whereas studies on the effects of life support training for any age group of patients have been conducted, their focus is mostly on knowledge and skill retention observed in simulated practice following course participation. Few studies have examined outcomes considered more meaningful such as morbidity, mortality or work-place provider practices [9–11,14,15,22].

Previously, video recording was used as a means of evaluating neonatal resuscitation performance of healthcare providers [18,19]. In particular, this approach allowed researchers to: 1) determine the actual conduct of neonatal resuscitation in a specific institution; 2) compare that resuscitation against the standards set forth by the American Heart Association guidelines for neonatal resuscitation (i.e. NRP program) [7]; and 3) re-educate and improve performance [18,19].

This study represents the first prospective analysis of neonatal resuscitation in a low-resource clinical setting. We objectively assessed the performances of midwives involved in neonatal resuscitation before and after participation in an adapted NRP training program. Similar to previous studies conducted in high-resource countries [18,19,23], we found several deviations from NRP guidelines.

Although midwives had a long experience (average 7 years) in the delivery room and had previously participated in a median of 2 adapted NRP courses, the percentage of correct procedures before the course was very low for all resuscitation interventions (initial steps, BMV and CC). After the course, we noted an overall improvement, with the most significant improvements noted for positioning of the head, drying, stimulation, starting ventilation with room air, and correct positioning of the face mask.

The opportunity to analyze the videos allowed us to assess in depth the three levels of resuscitation (initial steps, BMV and CC) and showed that the application of knowledge and skill retention in clinical practice depends on the specific intervention. For example, initiation of BMV with room air instead of 100% oxygen or the correct positioning of the face mask were easier to teach (and learn) than the correct ventilatory rate, effective chest movements and detection of the heart rate. On the contrary, the frequent and aggressive use of deep suctioning was clearly documented, with the course having no impact in changing this practice. These findings are difficult to interpret and need to be discussed in depth with participants to understand the reasons of “failure” and “success” of the course on clinical practice. Based on the results of the present study, we have planned a further educational intervention consisting in two phases: a) a local instructor holds weekly sessions on a manikin for delivery room healthcare providers; b) during the last week of the month, video cameras are switched on to collect data on resuscitation practices with the purpose of documenting the improvements and eventually provide a personalized learning curve. In this context, the information obtained through the use of video recordings can provide useful and objective feedback to both the teaching staff as well as the participants of the NRP course. This feedback helps in identifying the strengths and weaknesses of the educational intervention.

A further important finding of the present study concerns the times of resuscitation interventions. We found that the times of initiation and duration of all procedures were inconsistent with the times recommended by NRP algorithm [7]. Median times from birth to initiation of suction, heart rate detection, BMV and CC decreased in the period after the course, but remained longer than those recommended. This information should be discussed with the resuscitation team to understand the reasons for this delay. However, it must be noted that the poor performance of health care providers has multiple determinants [24].

In line with previous studies conducted in high-resource settings, it was found that the staff adapted very quickly to the presence of the recorder [19]. The system was very simple to use, unobtrusive and did not interfere with staff activity.

The strength of the present study is the objective assessment of healthcare worker performance in a low-resource delivery room through the use of video recording. Nevertheless, it has some limitations that should be considered when interpreting the results. A small number of participants were involved in the study, although they represented the entire staff involved in the care of the 4500 newborns born at Beira Central Hospital. Although this situation reflects a typical organizational and cultural environment of a referral African delivery setting, our results could be different in other contexts. The training was based on the NRP course significantly adapted to a low-resource setting; other educational initiatives such as “Helping Babies Breathe” program could have a different impact [25]. We chose to implement a modified NRP training program in this low-resource setting, rather than the Helping Babies Breathe curriculum because previous NRP courses were held in this hospital and because the trainers were certified as NRP instructors. However, with the exception of small differences such as the time of heart rate assessment, the algorithms are very similar between the two programs.

Previous studies have raised some concerns on the validity of video monitoring [26], however we believe that the limitations of this system (ie, single view of patient or inability to capture all resuscitations in all delivery rooms) are minor relative to the strengths of the system.

While there is increasing pressure to implement training programs on neonatal resuscitation in developing countries, it is important that their true effects on actual healthcare provider performance and neonatal morbidity and mortality are established. Such studies need to be based in typical, low income settings where supervision and opportunities for continuous learning or ongoing mentorship and resources for post-resuscitation care may be limited.

## Conclusions

The primary purpose of this study was to use video recording to evaluate the effect on healthcare provider performance of an adapted NRP course in a low-resource setting. Our results show that although resuscitations remained below the recommended standards in terms of quality and time of execution, clinical performance did show some improvement after the course. In addition, staff adapted very quickly to the presence of the recorder. Video recording is useful for objective assessment of staff performance during resuscitation and can be used as an educational tool. However, it remains to be established if it could be helpful to improve resuscitation practices in a low-resource delivery room. Our study highlights that the current practice may be insufficient to improve outcomes in this highly important clinical setting, and evaluation of further methods to improve this area of practice is warranted.

## Supporting Information

S1 Dataset Dataset. (PDF)

S1 Table Modified scoring system. (PDF)
