# Supplementary material for: Effect of a Neonatal Resuscitation Course on Healthcare Providers’ Performances Assessed by Video Recording in a Low-Resource Setting
Source: PLoS One. 2015 Dec 11;10(12):e0144443. doi: 10.1371/journal.pone.0144443 (PMC4684235; doi:10.1371/journal.pone.0144443)
Supplement: S1 Dataset — (PDF) [file pone.0144443.s001.pdf]

IDprocedure	Group	Performed Initial Steps	Equipment preparation	Newborn positioning	Head positioning	Suction	Drying	Remove wet linen	Naked newborn	Stimulation	HR assessment	Score Initial Steps	Performed BMV	Start PPV in room air	Correct FM positioning	Correct RR frequency	Chest movements	HR detection (after 30 sec)	Score BMV	Performed CC	Correct method	Correct frequency and depth	Correct ventilation frequency	Correct CC/ventilation ratio (3:1)	HR assessment	Score CC
1	BC	yes	0	0	2	1	0	0	0	1	0	22,22222222	yes	0	0	0	0	0	0	yes	0	0	0	0	0	0
2	BC	yes	0	2	2	1	1	0	0	0	0	33,33333333	no					0	0	no						0
3	BC	yes	2	2	2	0	0	0	0	1	0	38,88888889	yes	2	2	0	0	0	40	no						0
4	BC	yes	0	0	0	1	0	0	0	0	0	5,555555556	no					0	0	no						0
5	BC	yes	0	2	0	1	0	0	0	0	0	16,66666667	yes	2	0	0	0	2	40	no						0
6	BC	yes	0	2	2	1	0	0	0	0	0	27,77777778	no					0	0	no						0
7	BC	yes	0	2	2	1	1	0	0	0	0	33,33333333	no					0	0	no						0
8	BC	yes	0	2	0	1	0	0	0	0	0	16,66666667	yes	0	2	2	2	2	80	yes	0	0	0	0	2	20
9	BC	yes	0	2	2	1	1	0	0	1	0	38,88888889	yes	2	0	0	2	2	60	yes	0	0	0	0	0	0
10	BC	yes	0	2	2	1	0	0	2	1	1	50	yes	2	2	2	2	0	80	yes	0	0	0	0	0	0
11	BC	yes	0	2	2	1	0	0	0	0	0	27,77777778	yes	2	0	0	0	0	20	no						0
12	BC	yes	0	0	0	1	0	0	0	0	0	5,555555556	no					0	0	no						0
13	BC	yes	0	2	2	1	0	0	0	0	0	27,77777778	no					0	0	no						0
14	BC	yes	0	2	2	1	2	0	2	0	1	55,55555556	yes	2	0	0	0	0	20	no						0
15	BC	yes	2	2	0	1	0	0	0	1	0	33,33333333	yes	2	2	0	2	0	60	no						0
16	BC	yes	0	0	0	1	0	0	0	1	0	11,11111111	no					0	0	no						0
17	BC	yes	0	2	2	1	0	0	2	1	1	50	yes	0	0	0	0	0	0	no						0
18	BC	yes	0	2	2	1	1	0	0	1	0	38,88888889	no					0	0	no						0
19	BC	yes	0	0	0	1	0	0	2	0	1	22,22222222	no					0	0	no						0
20	BC	yes	0	2	2	1	0	0	0	0	0	27,77777778	yes	2	2	0	2	0	60	yes	0	0	0	0	0	0
21	BC	yes	0	2	0	1	1	0	2	0	0	33,33333333	no					0	0	no						0
22	BC	yes	0	2	2	1	0	0	0	1	1	38,88888889	yes	0	0	0	0	0	0	yes	0	0	0	0	2	20
23	BC	yes	2	2	0	1	0	0	2	0	1	44,44444444	yes	2	0	0	0	2	40	yes	0	0	0	0	2	20
24	BC	yes	0	2	2	1	0	0	0	1	1	38,88888889	yes	2	2	0	2	2	80	no						0
25	BC	yes	0	2	2	1	0	0	0	0	0	27,77777778	no					0	0	no						0
26	BC	yes	0	2	2	1	1	0	2	1	0	50	yes	0	0	0	0	0	0	no						0
27	BC	yes	0	2	2	1	2	2	2	0	0	61,11111111	no					0	0	no						0
28	BC	yes	0	2	2	1	2	0	2	1	1	61,11111111	yes	0	0	0	2	0	20	no						0
29	BC	yes	2	2	2	1	0	0	0	1	0	44,44444444	yes	2	2	0	0	0	40	yes	0	0	0	0	0	0
30	BC	yes	0	2	2	1	0	0	0	1	0	33,33333333	yes	0	2	0	0	0	20	no						0
31	BC	yes	0	0	0	1	2	0	0	1	1	27,77777778	yes	0	0	2	2	2	60	yes	0	0	0	0	0	0
32	BC	yes	0	2	2	1	0	0	0	0	1	33,33333333	yes	0	2	0	2	0	40	yes	2	0	0	0	2	40
33	BC	yes	0	2	2	1	0	0	0	0	0	27,77777778	yes	2	0	0	0	0	20	yes	0	0	0	0	0	0
34	BC	yes	2	2	2	1	0	0	0	1	0	44,44444444	yes	2	0	0	0	0	20	no						0
35	BC	yes	0	2	2	1	0	0	0	0	0	27,77777778	yes	0	0	0	0	0	0	yes	0	0	0	0	0	0
36	BC	yes	0	2	0	0	0	0	0	0	0	11,11111111	yes	0	0	2	0	0	20	yes	0	0	0	0	0	0
37	BC	yes	0	2	0	1	0	0	0	0	1	22,22222222	yes	2	0	0	0	0	20	yes	0	0	0	0	0	0
38	BC	yes	0	2	2	1	0	0	0	1	1	38,88888889	yes	0	2	0	0	0	20	yes	0	0	0	0	0	0
39	BC	yes	2	2	2	1	0	0	0	1	0	44,44444444	yes	2	2	0	0	0	40	no						0
40	BC	yes	0	2	2	1	0	0	0	1	0	33,33333333	yes	2	2	0	0	0	40	yes	0	0	0	0	0	0
41	BC	yes	0	2	2	1	1	0	0	1	0	38,88888889	yes	0	0	0	0	0	0	no						0
42	BC	yes	0	2	2	1	0	0	0	1	0	33,33333333	yes	0	0	2	0	0	20	no						0
43	BC	yes	0	2	2	1	0	0	2	1	0	44,44444444	yes	2	2	0	0	0	40	yes	2	0	0	0	0	20
44	BC	yes	0	2	2	1	0	0	0	1	0	33,33333333	yes	0	0	0	0	0	0	yes	0	0	0	0	0	0
45	BC	yes	0	2	2	1	0	0	0	1	0	33,33333333	no					0	0	no						0
46	BC	yes	0	2	2	1	0	0	0	0	1	33,33333333	yes	0	2	2	2	0	60	yes	0	0	0	0	0	0
47	BC	yes	0	2	2	1	0	0	0	1	0	33,33333333	yes	2	2	0	0	0	40	no						0
48	BC	yes	0	2	2	1	0	0	0	1	1	38,88888889	no					0	0	no						0
49	BC	yes	0	2	2	1	0	0	0	1	0	33,33333333	yes	0	0	0	0	0	0	no						0
50	BC	yes	0	2	2	1	0	0	0	1	0	33,33333333	yes	2	0	0	0	0	20	yes	0	0	0	0	0	0
51	AC	yes	0	2	2	1	2	0	0	0	0	38,88888889	no					0	0	no						0
52	AC	yes	0	2	2	1	2	0	0	1	0	44,44444444	no					0	0	no						0
53	AC	yes	0	2	2	1	0	0	0	1	2	44,44444444	yes	2	2	0	2	0	60	yes	2	2	2	2	2	100
54	AC	yes	0	2	2	1	0	0	0	1	1	38,88888889	yes	0	2	2	2	0	60	no						0
55	AC	yes	2	2	2	0	2	0	0	1	1	55,55555556	yes	2	2	0	2	0	60	yes	0	2	2	2	0	60
56	AC	yes	0	2	2	1	2	0	2	1	1	61,11111111	yes	2	2	0	0	0	40	no						0
57	AC	yes	0	2	2	1	0	0	0	1	1	38,88888889	yes	2	2	0	0	0	40	yes	2	0	0	0	0	20
58	AC	yes	0	2	2	1	2	0	0	1	0	44,44444444	yes	2	2	0	0	0	40	no						0
59	AC	yes	0	2	2	1	0	0	0	1	0	33,33333333	yes	0	2	2	2	0	60	no						0
60	AC	yes	2	2	2	1	2	0	0	2	0	61,11111111	yes	2	2	2	2	0	80	no						0
61	AC	yes	0	2	2	1	1	0	0	1	0	38,88888889	yes	2	0	0	0	0	20	no						0
62	AC	yes	2	2	2	1	0	0	2	0	1	55,55555556	yes	2	0	0	0	0	20	yes	0	0	2	2	0	40
63	AC	yes	2	2	0	1	1	0	0	1	1	44,44444444	yes	2	2	0	0	0	40	yes	2	0	0	2	0	40
64	AC	yes	2	2	2	0	1	0	0	1	1	50	yes	2	2	0	2	2	80	no						0
65	AC	yes	0	2	2	1	1	0	0	1	1	44,44444444	yes	2	2	0	2	0	60	no						0
66	AC	yes	0	2	2	1	0	0	0	1	0	33,33333333	yes	2	0	0	0	0	20	no						0
67	AC	yes	0	2	2	1	1	0	0	1	0	38,88888889	no					0	0	no						0
68	AC	yes	0	2	2	1	2	0	0	1	0	44,44444444	yes	2	2	2	2	0	80	no						0
69	AC	yes	2	2	2	1	2	0	2	1	0	66,66666667	no					0	0	no						0
70	AC	yes	0	2	2	0	2	0	0	1	1	44,44444444	yes	2	2	0	2	0	60	no						0
71	AC	yes	0	2	2	1	0	0	0	1	1	38,88888889	yes	0	2	0	2	0	40	yes	2	2	2	2	0	80
72	AC	yes	2	2	2	1	2	0	2	1	1	72,22222222	yes	2	2	2	2	0	80	no						0
73	AC	yes	0	2	2	1	0	0	0	1	0	33,33333333	yes	2	2	0	0	0	40	no						0
74	AC	yes	0	2	2	0	2	0	2	1	1	55,55555556	no					0	0	no						0
75	AC	yes	0	0	2	1	0	0	2	1	0	33,33333333	no					0	0	no						0
76	AC	yes	2	2	2	1	2	2	0	0	0	61,11111111	yes	2	2	2	2	0	80	no						0
77	AC	yes	0	2	2	1	2	0	0	1	1	50	yes	2	2	2	0	2	80	no						0
78	AC	yes	0	2	2	1	1	0	0	0	1	38,88888889	no					0	0	no						0
79	AC	yes	0	2	2	1	2	0	0	0	0	38,88888889	no					0	0	no						0
80	AC	yes	0	2	0	1	0	0	0	1	0	22,22222222	yes	2	2	0	0	0	40	no						0
81	AC	yes	2	2	2	1	0	0	2	1	1	61,11111111	yes	0	2	0	2	0	40	no						0
82	AC	yes	2	2	2	1	0	0	2	1	1	61,11111111	yes	2	2	2	2	0	80	no						0
83	AC	yes	2	2	2	1	0	0	0	1	0	44,44444444	yes	2	2	0	0	0	40	yes	0	0	0	0	0	0
84	AC	yes	0	2	2	1	1	0	2	1	1	55,55555556	yes	2	2	0	0	0	40	no						0
85	AC	yes	0	2	2	1	1	0	0	0	0	33,33333333	yes	2	2	0	0	0	40	no						0
86	AC	yes	0	2	2	1	0	0	0	0	1	33,33333333	yes	2	2	0	0	0	40	yes	0	0	0	0	2	20
87	AC	yes	0	2	2	1	1	0	0	1	0	38,88888889	yes	2	2	0	2	0	60	no						0
88	AC	yes	0	2	2	1	2	0	0	0	0	38,88888889	yes	2	2	0	0	0	40	no						0
89	AC	yes	0	2	2	1	2	0	0	0	0	38,88888889	yes	2	0	0	0	0	20	no						0
90	AC	yes	0	2	2	1	0	0	0	1	0	33,33333333	yes	2	2	0	0	0	40	no						0
91	AC	yes	0	2	2	1	0	0	0	1	0	33,33333333	yes	2	2	2	0	0	60	no						0
92	AC	yes	0	2	2	0	1	0	0	1	0	33,33333333	no					0	0	no						0
93	AC	yes	0	2	2	1	0	0	0	1	0	33,33333333	yes	2	2	0	0	0	40	no						0
94	AC	yes	2	2	2	1	0	0	2	1	0	55,55555556	yes	2	2	0	0	0	40	no						0
95	AC	yes	0	2	2	1	2	0	0	1	0	44,44444444	yes	2	2	0	0	0	40	no						0
96	AC	yes	2	2	2	1	1	0	0	1	0	50	yes	2	2	0	0	0	40	yes	0	0	0	0	0	0
97	AC	yes	2	2	2	1	0	0	2	1	0	55,55555556	yes	2	2	0	0	0	40	no						0
98	AC	yes	2	2	2	1	0	0	0	1	0	44,44444444	yes	2	2	0	2	0	60	yes	0	0	0	0	0	0
99	AC	yes	0	2	2	1	0	0	0	1	0	33,33333333	yes	2	0	0	0	0	20	yes	2	0	0	0	0	20
100	AC	yes	0	0	2	1	0	0	2	1	1	38,88888889	yes	2	2	0	0	0	40	yes	0	0	0	0	0	0
