# Supplementary material for: Effect of a Neonatal Resuscitation Course on Healthcare Providers’ Performances Assessed by Video Recording in a Low-Resource Setting
Source: PLoS One. 2015 Dec 11;10(12):e0144443. doi: 10.1371/journal.pone.0144443 (PMC4684235; doi:10.1371/journal.pone.0144443)
Supplement: S1 Table — (PDF) [file pone.0144443.s002.pdf]

ID of patient: …………….

Evaluation date: …………..

EVALUATION SCORE: - 2 = correctly done; 0 = not done; 1 = done, but incorrect for method and/or timing

INITIAL STEPS

Score	2	0	1	Time elapsed from birth/comments
Check material (suction system, bag, mask, oxygen supply)				
Body positioned correctly				
Head positioned correctly				
Suctioning (if indicated)				
Dry the baby				
Remove wet linen				
Neonate uncovered under infant warmer				
Stimulation				
HR assessment (stethoscope or umbilical pulse)				
**TOTAL**				

VENTILATION

Score	2	0	1	Time elapsed from birth/comments
Start PPV in room air				
Correct face mask positioning				
Ventilatory rate at 40-60 breaths/min				
Adequate chest rise				
HR assessment after 30 sec (stethoscope or umbilical pulse)				
**TOTAL**				

CHEST COMPRESSIONS

Score	2	0	1	Time elapsed from birth/comments
Correct method (2 fingers/2 thumbs)				
Correct frequency and depth				
Correct ventilatory frequency				
Correct CC/ventilation ratio (3/1)				
HR assessment after 60-90 sec (stethoscope or umbilical pulse)				
**TOTAL**				
